# Selective COX-2 inhibitors do not increase gastrointestinal reactions after colorectal cancer surgery: a systematic review and meta-analysis

**DOI:** 10.1186/s12876-023-02918-w

**Published:** 2023-08-14

**Authors:** Ting Hu, Cheng-Jiang Liu, Xiaoming Yin, WenJuan Tang, LanFang Yin, Hui Bai, FangFang Liu, Dan Wang, YiLei Li

**Affiliations:** 1 Department of General Practice, Anqing Municipal Hospital, Anqing, 246000 AnHui China; 2 Department of General Medicine, Affiliated Anqing First People’s Hospital of Anhui Medical University, Anqing, 246000 AnHui China

**Keywords:** Colorectal cancer, Chemoprevention, COX-2 inhibitor, Celecoxib, meta-analysis

## Abstract

**Background:**

The effectiveness of selective COX-2 inhibitors in preventing colorectal cancer recurrence has been demonstrated, however it is unknown how safe and successful they will be over the long term. As a result, we looked at the efficacy, safety, and consequences of adding COX-2 inhibitors to the treatment plan afterward.

**Methods:**

In patients with advanced colorectal cancer, we compared the efficacy of celecoxib at two different doses (200 mg twice day and 400 mg twice daily) with placebo. To evaluate the impacts of post-treatment, several datasets from inception to June 2022 were searched. Response rate, illness control rate, and 3-year survival were the main results. And evaluated several safety outcomes, particularly those that were susceptible to adverse events.

**Results:**

The study comprised a total of 9 randomized controlled trials (3206 participants). Celecoxib and rofecoxib doidn’t significantly improved the 1–3 year remission rate (OR, 1.57 [95% CI: 0.95–2.57]) and disease control rate (OR, 1.08 [95% CI: 0.99–1.17]). Subgroup analysis of different doses showed that 400 mg of celecoxib significantly improved the response rate (OR, 2.82 [95%CI: 1.20–6.61]). 200 mg celecoxib was not significant (OR, 1.28 [95% CI: 0.66–2.49]). Rofecoxib also did not fully improve disease response rates. Celecoxib at any dose improved 3-year survival (OR, 1.21 [95% CI: 1.02–1.45]). It is important to note that COX-2 inhibitors did not significantly enhance the likelihood of adverse events including gastrointestinal or cardiovascular side effects at any dose.

**Conclusions:**

For patients with advanced colorectal cancer, a reasonable chemoprevention regimen can include celecoxib 400 mg twice daily.

**Supplementary Information:**

The online version contains supplementary material available at 10.1186/s12876-023-02918-w.

## Introduction

In 2018, colorectal cancer (CRC) was the second leading cause of cancer-related death worldwide. In 2018, CRC is anticipated to have been directly responsible for around 244,000 deaths in Europe [1]. Surgery is a highly effective treatment option when a disease is localized to one area of the body. When CRC has progressed to a later stage, it can be difficult to find a treatment plan that has the intended superior effect. Chemotherapy for patients with advanced CRC commonly consists of 5-fluorouracil (5-FU), leucovorin (LV), oxaliplatin, or irinotecan (FOLFOX or FOLFIRI) [2, 3]. Survival rates, quality of life, and the number of patients requiring secondary operations are all factors in the growing importance of optimizing first-line treatment plans.

Prostaglandin production relies heavily on the cyclooxygenase-2 (COX-2) enzyme, which is highly expressed in inflammatory and tumor tissues [4, 5]. COX-1, COX-2, and COX-3 are all COX isomers, however they all have different purposes in the body. It is a universally accepted fact that human cells and tissues always and everywhere express the maintenance enzyme COX-1. However, the splice variant of COX-1 known as COX-3 is expressed in humans but serves no biological purpose [6]. COX-2 is a pro-inflammatory enzyme that is strongly linked to inflammatory diseases. Angiogenesis, tumor tissue invasion, and resistance to apoptosis are all caused by the inducible enzyme COX-2, which has also been linked to inflammatory conditions and carcinogenesis. Consequentially, the COX-2 and prostaglandin cascades play a significant role in the “inflammation of cancer” [7, 8, 9, 10].

90% of lung cancers, 71% of colon cancers, and 56% of breast malignancies have been found to express COX-2 at moderate to high levels [11, 12, 13]. Using multivariate analysis, researchers found that elevated COX-2 levels independently predicted worse outcomes for cancer patients [14]. Multiple lines of evidence suggest that the cyclooxygenase-2 (COX2) enzyme plays a role in the development and progression of colorectal cancer [15]. COX2 has been shown to promote growth, migration, and invasiveness; inhibit apoptosis; and boost angiogenesis. Celecoxib, rofecoxib, etoricoxib, nabumetone, meloxicam, and etodolac, among others, were associated with a reduced risk of death in a population-based retrospective cohort analysis [16]. Consistent aspirin use in conjunction with any dose of COX-2 inhibitors has also been shown to lower mortality and recurrences in colon cancer [17].

Celecoxib is an essential component of tumor therapy because it inhibits the proliferation of tumor cells by promoting apoptosis and shifting the cell cycle. Selective COX-2 inhibitors have been found to increase life expectancy and enhance quality of life when used in conjunction with standard medical care. Therefore, we performed a meta-analysis and a comprehensive literature search to ascertain the impact of drug addition on patient outcomes.

## Methods

This study was reported in accordance with the Preferred Reporting Items for Systematic Reviews and Meta-Analysis (PRISMA) standard.

### Search strategy

We found relevant studies after conducting a complete search of PubMed, Medline, and EMBASE up until June 2022. Also, we looked for new research by reading through older systematic reviews. Further information regarding the search strategy is provided in Table 1. The following inclusion criteria were met by randomized controlled trials (RCTs) and long-term follow-up of that: Celecoxib at any dose was the intervention; the placebo or control group was the comparison group; and the number of patients who acquired colorectal neoplasms was the outcome. Individuals with a higher risk of adenomas (≥ 18 years old) participated in the study. Every participant underwent a polypectomy and shown that their colon was polyp-free before they were assigned to a group. All of the participants had a history of adenomas.

**Table 1:** Characteristics of included studies

Study	Location	Phase	Study period	Mean age (Year)	ECOG or WHO PS	Sample size (Number of case/control)	Co-treatment regimen	Celecoxib treatment program (Drugs/dosage (mg/m2)/d/frequency of cycles)
Jin 2011	China	II	2005.6-2008.1	-	0–2	88(58/30)	folinic acid + fluorouracil + oxaliplatin	200 mg twice daily no fewer than 8 weeks
Köhne 2007	USA	II	2002.6-2005.11	about 70	0–2	44(23/21)	irinotecan + FA + 5-FU	200 mg twice daily,800 mg
	USA	II	2002.6-2005.11	about 70	1–2	41(19/22)	irinotecan + capecitabine	200 mg twice daily,800 mg
Maiello 2006	Italy	II	2003.1-2004.12	64	0–2	81(41/40)	irinotecan + FA + 5-FU	400 mg twice daily,repeated every 2weeks
Haldar 2020	USA	II	-	58	0–2	34(16/18)	etodolac + propranolol	400 mg twice daily
Fenwick 2003	UK	II	2000.12-2002.2	about 65	0–2	44(23/21)	placebo	25 mg twice daily,rofecoxib
Mostafa 2022	Egypt	II	2018.10-2020.7	about 44	0–2	54(26/28)	irinotecan + FA + 5-FU	200 mg twice daily last 3 months
Niu 2010	China	II	2006.1-2008.12	56	0–1	60(30/30)	irinotecan	200 mg twice daily
Li 2018	China	II	2015.2-2016.11	63	0–2	122(61/61)	oxycodone hydrochloride	200 mg twice daily last 1 months
Meyerhardt 2021	USA	III	2010.6-2015.11	61	0–2	2524(1263/1261)	folinic acid + fluorouracil + oxaliplatin	400 mg twice daily
Debucquoy 2009	USA	II	-	-	0–2	80(35/45)	CRT + 5-FU	400 mg twice daily
Hu 2022	China	II	2019.5-2021.4	18–75	0–2	34(17/17)	toripalimab	200 mg twice daily last 2 weeks

Note:FA = folinic acid, 5-FU = 5-fluorouracil, CRT = Chemotherapy treatment

### Outcomes of interest

One of the most notable effectiveness results that stood out to people was the number of colorectal adenomas that returned more than once (advanced adenomas and any adenomas). Each of the following was true of advanced adenomas: a diameter of at least one centimeter; villous or tubulovillous histology; high-grade dysplasia; intramucosal carcinoma; invasive malignancy. On the spectrum of adenoma development, from benign to malignant, we find invasive adenomas (classified as one or two small [1 cm] tubular adenomas or serrated polyps without cytologic dysplasia). Colorectal cancer rates, all-cause mortality rates, serious adverse events, cardiovascular disease rates, kidney disease rates, blood pressure levels, and any reports of post-randomization follow-up were also considered. The study authors determined that adverse events were considered serious if they led to death, hospitalization, severe gastrointestinal bleeding, cardiovascular problems, or the cessation of an intervention. Cardiovascular death, myocardial infarction, stroke, heart failure, and thromboembolic event were all categorized as “serious cardiovascular events” by the study’s authors. Renal and hypertensive effects include elevated blood creatinine levels, fluid retention and edema, hypertension, proteinuria, and renal failure. On top of that, hypertension has been linked to at least a few deaths in recent history. We analyzed the effects of celecoxib on the recurrence of colorectal neoplasia in patients who had been on the drug for at least two years after treatment had ended.

### Data extraction and quality assessment

Two reviewers reviewed the primary papers and then used a standardized form to record data on the study, the participants, and the treatment. There was no more confusion after the group discussion than there had been before it. The efficacy results were gathered using an updated version of the intention-to-treat analysis (i.e., subjects who received at least one dose of celecoxib at any dose and had at least one colonoscopy after randomization). The data on safety outcomes were compiled and analyzed using the intention-to-treat principle and the original trial participants who were randomly allocated to each study arm. Those who were unable to be reached for further evaluation were found to have experienced no negative outcomes. The Rob2.0 tool for evaluating the quality of randomized parallel and crossover trials was used for this study. This tool includes the following content: randomization/allocation process, deviations from expected interventions, missing outcome data, outcome measurement, and selective outcome reporting [18, 19]. By reviewing the papers until a consensus was reached, the reviewers were able to address their concerns about the inclusion of certain studies, the collection and processing of data, and the appraisal of the potential for bias.

### Data synthesis and statistical analysis

Review Manager’s meta-analysis made use of the random-effects model developed by DerSimonian and Laird. Therefore, we were able to estimate pooled risk ratios and 95% confidence intervals that took into account heterogeneity both within and between trials. We used I2 statistics to assess whether or not primary outcomes varied significantly across trials, with values above 50% indicating significant heterogeneity. Due to the small sample size, it was not able to determine whether or not publication bias had occurred. As a result, it became more difficult to determine whether or not an observed disparity was the product of a genuine cause or a mere coincidence. Subgroup analysis was performed with respect to the different celecoxib dosages, including 200 mg twice day (400 mg/day) and 400 mg twice daily (800 mg/day). Sensitivity analysis was also carried out to further ensure the reliability of the findings.

## Result

### Characteristics of included studies

**Fig. 1:**
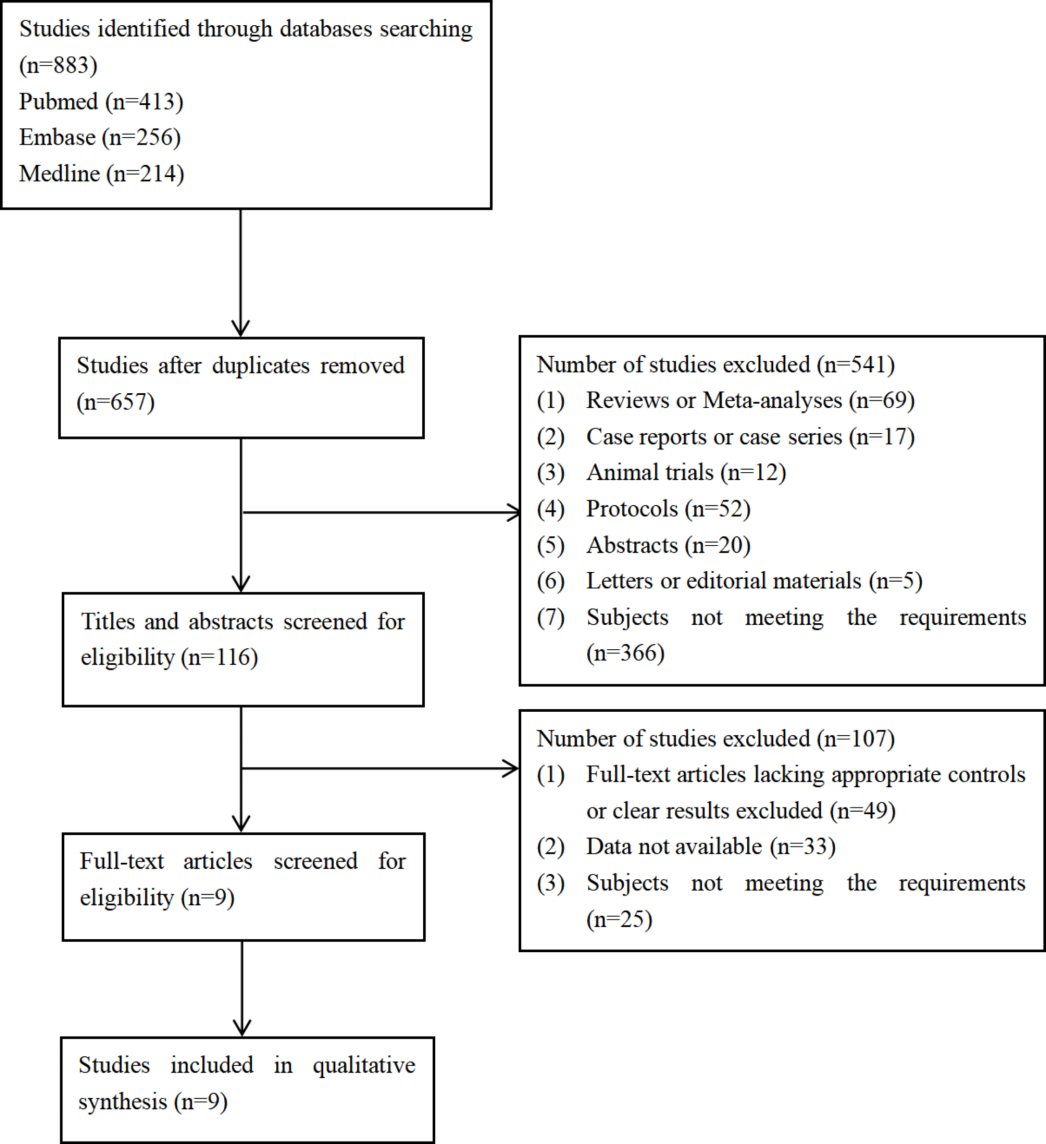
Screening flow chart of included literature

Figure 1 shows the screening process of this study. A total of 9 articles [20, 21, 22, 23, 24, 25, 26, 27, 28] were included for data extraction and analysis. Table 1 describes the characteristics of 9 RCTs. A total of 3206 patients were recruited in nine studies. The gender distribution included in the assessment was equal. Of these, 1594 patients received standard of care or placebo regimens, main drugs are folinic acid + fluorouracil + oxaliplatin, irinotecan + FA + 5-FU, oxycodone hydrochloride or others. And 1612 received supplemental COX-2 inhibitor therapy (Celecoxib), the frequency of cycles is 200 mg (7 articles) or 400 mg (4 articles) twice daily for more than 2 weeks. In another study, the drug regimen was 25 mg rofecoxib twice daily. 9 studies were of good quality according to the quality assessment of the modified Jadad scale. In studies with lower scores, random number generation was not reported, nor did they report on how randomization was concealed.

#### Quality assessment

Supplement S1 indicates the risk bias of the included studies. All studies did not provide a specific allocation plan, but were considered to be of high quality.

### Effects on the primary efficacy outcomes

#### Benefit of response rate(RR)

**Fig. 2:**
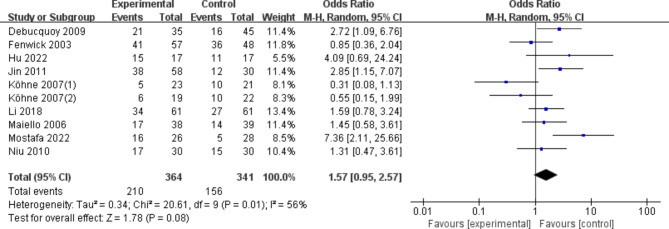
OR of COX-2 inhibitors compared to placebo in CRC treatment

According to the results of the meta-analysis, the use of any dose (200 mg or 400 mg of celecoxib) or rofecoxib did not statistically significantly improve response in patients with advanced CRC compared with placebo or control regimens (OR, 1.57 [95% CI: 0.95–2.57], Fig. 2). In the sensitivity analysis, the results were consistent with the primary analysis. Excluding studies using rofecoxib, the results were still robust (OR, 1.70 [95%CI: 0.99–2.90]). Using dose as a grouping criterion, the use of 200 mg of celecoxib resulted in the same results (OR, 1.28 [95% CI: 0.66–2.49]), and the use of 400 mg of celecoxib significantly improved the response of patients with advanced CRC (OR, 2.82 [95%CI: 1.20–6.61]).

#### Benefit of disease control rate(DCR)

**Fig. 3:**
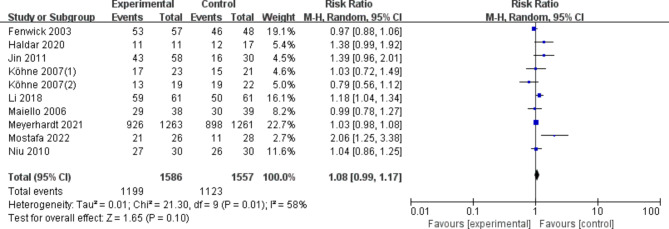
DCR of COX-2 inhibitors compared to placebo in CRC treatment

According to the results of the meta-analysis, the use of any dose (200 mg or 400 mg of celecoxib) or rofecoxib did not statistically significantly improve disease control rates in patients with advanced CRC compared with placebo or control regimens (OR, 1.08 [95%CI: 0.99–1.17], Fig. 3). In the sensitivity analysis, the results were consistent with the primary analysis. Excluding studies using rofecoxib, the results were still robust (OR, 1.11 [95%CI: 0.99–1.23]). Using dose as a grouping criterion, the same results were obtained with celecoxib 200 mg (OR, 1.19 [95% CI: 0.95–1.49]) and celecoxib 400 mg (OR, 1.09 [95% CI: 0.95–1.25]).

### Benefit of survival and quality of life improvement

**Fig. 4:**
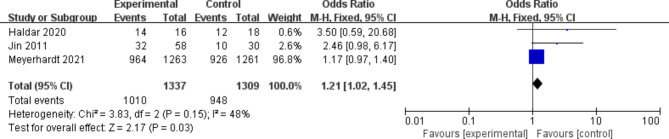
3-year survival of COX-2 inhibitors compared to placebo in CRC treatment

According to the results of the meta-analysis, the use of any dose (200 mg or 400 mg of celecoxib) statistically significantly improved 3-year survival in patients with advanced CRC compared with placebo or control regimens (OR, 1.21 [95% CI: 1.02–1.45], Fig. 4). In the sensitivity analysis, the results were consistent with the primary analysis. Using dose as a grouping criterion, the same results were obtained with celecoxib 200 mg (OR, 2.46 [95% CI: 0.98–6.17]) and celecoxib 400 mg (OR, 1.18 [95% CI: 0.99–1.41]) The results did not improve the patient’s condition. What is more significant is that the improvement rate of the quality of life of the patients in the control group is 40%, while the improvement rate of the patients in the experimental group is as high as 66%, and various physiological functions have recovered to a certain extent.

### Effects on the primary safety outcomes

**Fig. 5:**
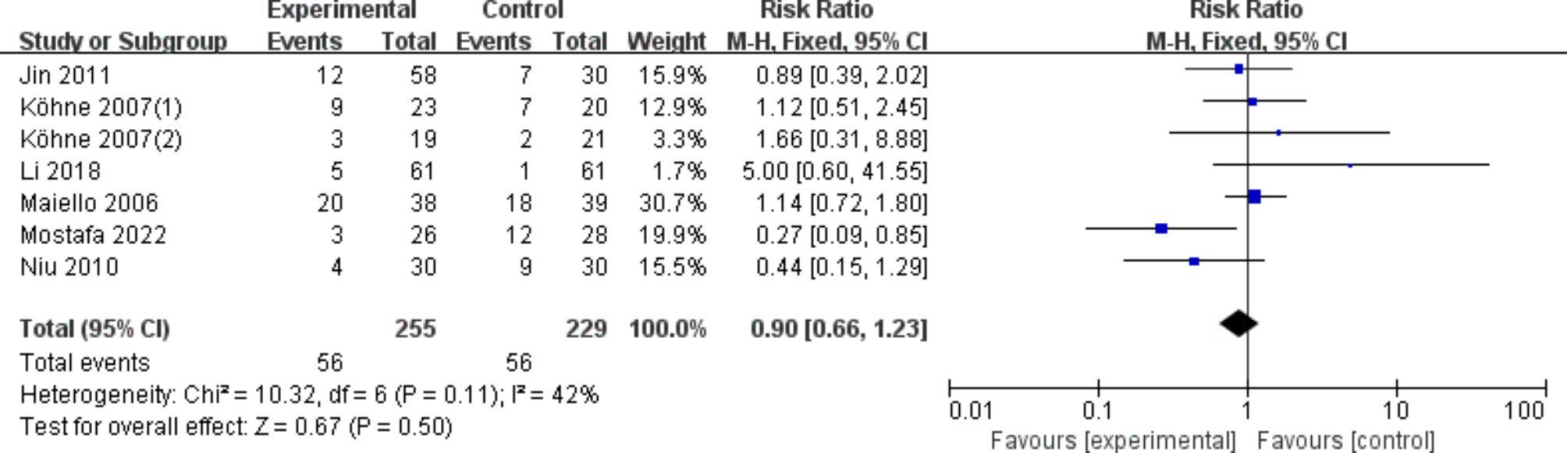
Nausea/vomiting of COX-2 inhibitors compared to placebo in CRC treatment

**Fig. 6:**
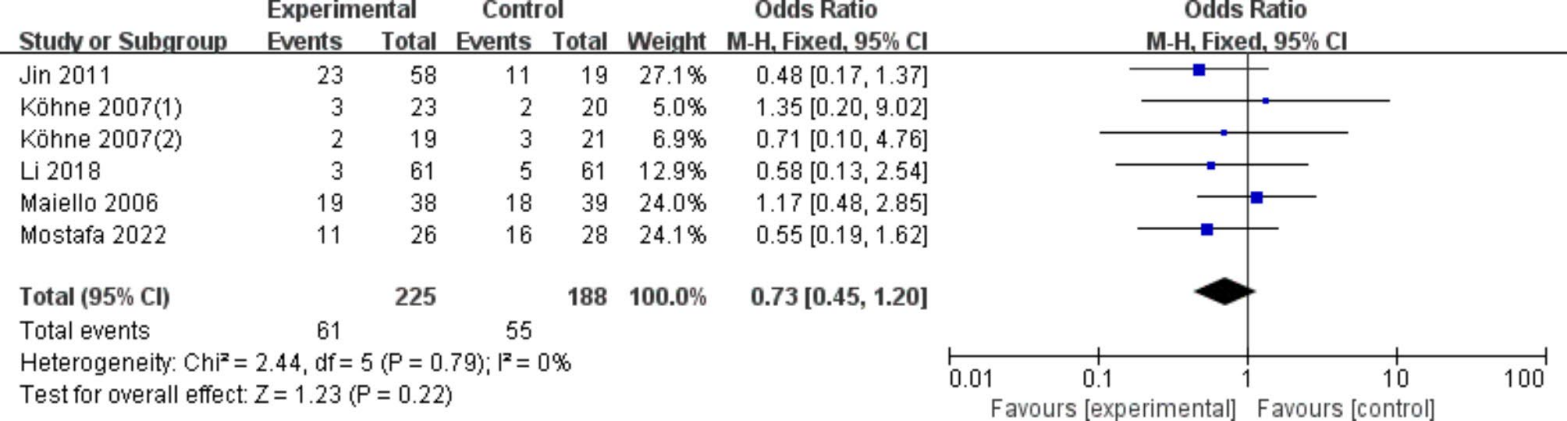
Diarrhoea of COX-2 inhibitors compared to placebo in CRC treatment

**Fig. 7:**
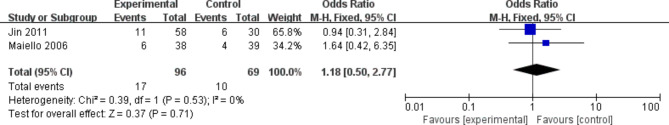
Oral mucositis of COX-2 inhibitors compared to placebo in CRC treatment

According to the results of the meta-analysis, the use of either dose (200 mg or 400 mg of celecoxib) did not statistically significantly improve the nausea/vomiting response rate (OR, 0.73 [95% CI: 0.45–1.20], Fig. 5) compared with placebo or control regimens. In the sensitivity analysis, the results were consistent with the primary analysis. Using dose as a grouping criterion, the same results were obtained with celecoxib 200 mg (OR, 0.62 [95%CI: 0.30–1.26]) and celecoxib 400 mg (OR, 0.86 [95% CI: 0.43–1.70]). There was no statistically significant increase in the incidence of diarrhea with either dose (200 mg or 400 mg of celecoxib) compared with placebo or control regimens (OR, 0.90 [95% CI: 0.66–1.23], Fig. 6). In In the sensitivity analysis, the results were consistent with the preliminary analysis. Using dose as a grouping criterion, the same results were obtained with celecoxib 200 mg (OR, 1.00 [95% CI: 0.64–1.58]) and celecoxib 400 mg (OR, 0.80 [95% CI: 0.52–1.21]). The use of either dose (200 mg or 400 mg of celecoxib) did not statistically significantly increase the incidence of Oral mucositis (OR, 1.18 [95% CI: 0.50–2.77], Fig. 7) compared with placebo or control regimens. Using dose as a grouping criterion, the same results were obtained with celecoxib 200 mg (OR, 0.94 [95% CI: 0.31–2.84]) and celecoxib 400 mg (OR, 1.64 [95% CI: 0.42–6.35]). In addition, neurotoxicity, Myelosuppression, etc. showed no significant difference.

## Discussion

COX-2 drugs are considerably protective against CRC recurrence, according to prior meta-analyses [29, 30, 31, 32, 33]. The nature of each COX-2 inhibitor and the intricate interactions between dose and baseline have prevented broader attention, even if the data from numerous studies are encouraging, particularly the rise in adverse effects. One of the few selective COX-2 inhibitors studied in numerous CRC preventive studies is celecoxib. Further research on his advantages is also made possible by the extensive application.

The preferred technique for preventing sporadic CRC is screening colonoscopy with removal of adenomatous polyps because it is associated with lower mortality [34, 35]. Additionally, despite routine screening, a small percentage of persons still develop CRC before the advised interval for surveillance, presumably as a result of missed or insufficient polyp removal or quickly growing tumors [36, 37]. COX-2 inhibitors are significant possibilities for easing the burden of CRC, according to numerous research.

The results of the initial randomized controlled trial and other meta-analyses are expanded upon in our study. The probability of advanced metachronous cancers was dramatically decreased by COX-2 inhibitors, particularly celecoxib. We discovered that in individuals with high-risk tumors at baseline, the advantages of COX-2 inhibitors may outweigh the risks of significant side effects. The long-term cardiovascular safety of COX-2 inhibitors is of concern, in addition to the short-term risk of major side events that we saw in our meta-analysis [38]. It’s important to note that such incidents are mostly observed in populations with a history of cardiovascular risk factors or disease. Conventional NSAIDs may not be linked to an elevated cardiovascular risk, according to recent literature [39]. Therefore, in populations with a low baseline risk of cardiovascular disease and an intermediate-high baseline risk of CRC, COX-2 inhibitors may be thought of as chemopreventive medicines.

The most popular COX-2 inhibitors in the FOLFIRI regimen, celecoxib and rofecoxib, were observed to significantly improve ORR and DCR in this study. The response rate shown a rise that was statistically significant, particularly in the short-term efficacy. These results are in line with a prior study that found patients with locally advanced rectal cancer who received celecoxib in combination to preoperative chemoradiation experienced an increase in excellent response [40, 41, 42]. The fact that celecoxib was well tolerated and there was no discernible difference in the frequency of adverse effects between the two groups is more concerning. Celecoxib was well tolerated at both higher dosages (800 mg/day) and lower doses (400 mg/day), according to earlier clinical investigations in CRC patients. Additionally, the current data are consistent with earlier studies that suggested celecoxib with chemotherapy did not increase toxicity in comparison to placebo [43, 44].

The serum VEGF levels were markedly lowered in the celecoxib/FOLFIRI group [45]. Celecoxib inhibits angiogenesis by lowering VEGF levels with the use of FOLFIRI and celecoxib. According to reports, celecoxib inhibits NF-kB by boosting the IkB inhibitor protein, lowering blood levels of CXCL5, and blocking the AKT/NF-kB pathway that is involved in cancer and angiogenesis [46]. Our positive findings regarding celecoxib’s impact on CXCL5 are in line with earlier research showing that increased CXCL5 levels are linked to colorectal metastases and a bad prognosis. Celecoxib and other selective COX-2 inhibitors have both shown antiangiogenic effects in a variety of in vitro and in vivo models [47, 48]. Selective COX-2 drugs have antiangiogenic action, however the exact mechanism is unknown. Endothelial cells may directly respond to selective COX-2 inhibitors [49, 50]. Due to CRC metastases, rofecoxib medication was linked to a 60% decrease in in vitro PGE2 synthesis. In fact, early results from a phase II trial involving celecoxib in combination with a variety of chemotherapy treatments for patients with metastatic colorectal cancer suggest that this strategy merits a controlled clinical examination.

However, this study has some limitations. Firstly, due to limited literature and research methods, it is not possible to conduct all subgroup analyses based on different drugs and doses. Secondly, it is difficult to unify dose equivalents across different trials and treatment plans. Although we attempted to standardize this definition during the data extraction process, it may not be fully effective. Third, the research on adding COX-2 in the treatment scheme is really lacking, and more high-quality randomized Scientific control are still needed.

### Electronic supplementary material

Below is the link to the electronic supplementary material.

**Supplement S1** Risk of bias graph and risk of bias summary.

## Data Availability

The datasets used and/or analysed during the current study available from the corresponding author on reasonable request.
