# Supplementary figures and images for: Selective COX-2 inhibitors do not increase gastrointestinal reactions after colorectal cancer surgery: a systematic review and meta-analysis
Source: BMC Gastroenterol. 2023 Aug 14;23:281. doi: 10.1186/s12876-023-02918-w (PMC10426080; doi:10.1186/s12876-023-02918-w)

Supplement S1 Risk of bias graph and risk of bias summary


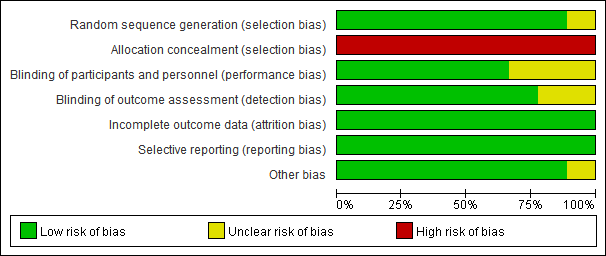


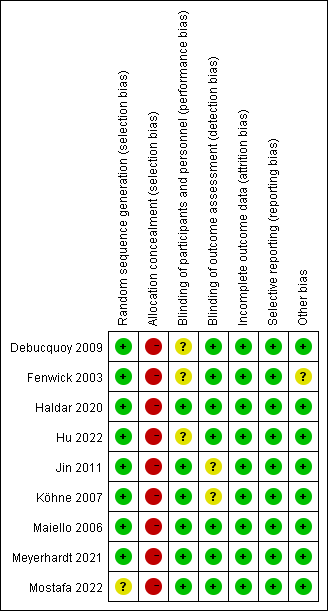

Supplement: Supplementary file 1 — Supplement S1 Risk of bias graph and risk of bias summary. [file 12876_2023_2918_MOESM1_ESM.docx]
